# Expression of glucosylceramide synthase in invasive ductal breast cancer may be correlated with high estrogen receptor status and low HER-2 status

**DOI:** 10.1186/1746-1596-9-22

**Published:** 2014-01-23

**Authors:** Jiannan Liu, Ping Sun, Yuan Sun, Aina Liu, Dong You, Fenge Jiang, Yuping Sun

**Affiliations:** 1 Department of Oncology, Jinan Central Hospital, Affiliated to Shandong University, Jinan, Shandong 250013, P R China; 2 Department of Oncology, Yuhuangding Hospital, Yantai, Shandong 264000, P R China; 3 Department of Ophthalmology, Zhongshan School of Medicine, Guangdong 510080, P R China

**Keywords:** Glucosylceramide synthase, Breast cancer, Multidrug resistance, ER, HER-2

## Abstract

**Abstract:**

**Background and objectives**

Breast cancer is one of the most common causes of cancer-related deaths in women worldwide. Studies on glucosylceramide synthase (GCS) activity suggest that this enzyme has a role in the development of multidrug resistance in many cancer cells. However, few studies have shown the expression of GCS in invasive ductal breast cancer and breast intraductal proliferative lesions.

**Methods**

In total, 196 samples from patients with invasive ductal breast cancer and 61 samples of breast intraductal proliferative lesions were collected. Immunohistochemical analyses were conducted to determine the expression of GCS and other related proteins.

**Results**

Expression of GCS was high in estrogen receptor (ER)-positive and HER-2 negative samples. In contrast, the expression of GCS in invasive ductal cancer was significantly lower than that in intraductal proliferative lesions.

**Conclusion**

Our data demonstrates a correlation between the expression of the GCS protein and ER-positive/HER-2 negative breast cancer. Furthermore, in contrast to previous reports, the expression of GCS protein was shown to be much higher in ductal carcinoma in-situ than that in invasive ductal cancer.

**Virtual slides:**

The virtual slide(s) for this article can be found here: http://www.diagnosticpathology.diagnomx.eu/vs/1559854430111589.

## Background

Breast cancer is one of the most common causes of cancer deaths in women worldwide [1]. The prognosis of breast cancer has been improved significantly by comprehensive therapy including surgical methods, chemotherapy, endocrine therapies and molecular targeted therapy. However, multidrug resistance (MDR) has been a major barrier to improved survival rates among breast cancer patients. MDR refers to the resistance of tumors not only to individual cytotoxic drugs used in chemotherapy, but also to cross-resistance to a range of drugs with different structures and cellular targets [2].

P-glycoprotein (P-gp, P170), encoded by the MDR1 gene (*ABCB1*) in humans is the major cause of multidrug resistance in breast cancer. P-gp is a member of the adenosine triphosphate-binding cassette (ABC) superfamily of membrane transporters, which bind and hydrolyze ATP. The energy produced in this reaction is used to drive the active transport of various molecules across the plasma membrane or the intracellular membranes of organelles, such as the endoplasmic reticulum, peroxisomes, and mitochondria. A wide range of anticancer agents are actively extruded by P-gp, leading to chemoresistance [3].

Many studies have indicated that MDR1 is regulated by glucosylceramide synthase (GCS), which is a pivotal enzyme in the regulation of cellular ceramide [4]. Studies on GCS activity suggest that the enzyme plays a role in the development of MDR in many cancer cells [5,6]. A number of methods that suppress the expression of GCS, such as specific inhibitors, antisense oligonucleotides and siRNA, have been shown to render MDR cells chemosensitive [7,8]. Gouaze et al. suggested that GCS blockade resensitizes MDR breast cancer cells to anticancer drugs by downregulation of P-glycoprotein [9]. Liu et al. further demonstrated that GCS upregulates MDR1 expression to regulate cancer drug resistance through cSrc and beta-catenin signaling [10].

Few studies have shown the expression of GCS in breast cancer tissue samples. In 2009, Ruckhäberle et al. analyzed microarray data that showed GCS mRNA expression levels in 1,681 breast tumors [11], but few data have demonstrated the expression of the GCS protein in breast cancer. In 2011, Liu et al. detected GCS expression levels in normal tissues and certain cancer tissues; however, this investigation was conducted in only a small number of samples [12]. Zhang et al. showed the relationship between chemotherapy and GCS expression in invasive breast cancer tissue. However, there are currently no reports describing the expression of GCS in clinical samples of intraductal proliferative breast lesions. This study aimed to rectify this omission.

## Methods

### Clinical samples

Tissue samples from 257 patients who underwent complete dissection of the breast and axillary lymph nodes (breast cancer patients) or local lumpectomy (patients with intraductal proliferative lesions or patients with accessory breast) were collected at the Qilu Hospital and Provincial Hospital, Shandong University, China, between January 2006 and June 2010. No patients had preoperative chemotherapy and informed consent for pathological evaluation was obtained from all patients prior to surgery.

Paraffin-embedded tumor samples were prepared from 196 patients with invasive ductal breast carcinoma, 25 patients with ductal carcinoma in-situ (DCIS), 11 patients with atypical ductal hyperplasia (ADH), 25 patients with usual ductal hyperplasia (UDH) and five patients with accessory breast. Histopathologic variables, including tumor size, lymph node metastasis, histologic subtype, and histologic grade, were determined by reviewing pathology reports and hematoxylin and eosin stained (H&E) sections. Patient and tumor characteristics are summarized in Tables 1 and 2. Forty of the ductal breast carcinoma patients received clinical follow-up at a median of 63 months (range, 15–68 months).

**Table 1:** Patient and tumor characteristics for the 143 reference invasive ductal breast cancer data series

**Characteristics**	**Number of patients**	**(%)**
Age (years)		
<35	8	(5.60)
35-60	108	(75.5)
>60	27	(18.9)
Tumor stage		
T1-2	131	(91.6)
T3-4	12	(8.40)
Nodal stage		
N0	72	(50.3)
N1-x	71	(49.7)
Histologic grade		
Grade I	16	(17.2)
Grade II	99	(69.2)
GradeIII	28	(19.6)
ER		
Negative	51	(35.7)
Positive	92	(64.3)
PR		
Negative	52	(36.4)
Positive	91	(63.6)
HER-2		
Negative	104	(72.7)
Positive	39	(27.3)
Ki67		
<14%	30	(18.2)
≥14%	113	(81.8)

ER, estrogen receptor; PR, progesterone receptor; HER-2, human epidermal growth factor-2.

**Table 2:** Patients and tumor characteristics for the 25 reference breast cancer in-situ data set (n = 25)

**Characteristics**	**Number of patients**	**(%)**
Age (years)		
<35	1	(4.0)
35-60	15	(60.0)
>60	9	(36.0)
Tumor stage		
T1-2	17	(69.2)
T3	4	(15.4)
Tx	4	(15.4)
Nodal stage		
N0	24	(96.2)
N1-x	1	(3.80)
ER		
Negative	7	(28.0)
Positive	18	(72.0)
PR		
Negative	5	(25.0)
Positive	20	(75.0)
HER-2		
Negative	19	(76.0)
Positive	6	(24.0)
Ki67		
<14%	16	(64.0)
≥14%	9	(36.0)

ER, estrogen receptor; PR, progesterone receptor; HER-2, human epidermal growth factor-2.

The use of these tissues was approved by the Research Ethics Committee of Shandong Medical University and we obtained written informed consent from all participants involved in our study.

### Cell culture

The multidrug-resistant breast cancer cell line, MCF-7/ADM, was selected from the drug sensitive breast cancer cell line MCF-7 using Doxorubicin in stages. Cells were maintained in RPMI 1640 medium supplemented with 10% (v/v) fetal bovine serum (FBS) in a humidified atmosphere containing 5% CO_2_ at 37°C. Cells were then seeded on glass slides for 24 h. Overexpression of GCS protein by MCF-7/ADM cells was confirmed for use of these cells as a positive control in this study [13].

### Morphologic parameters

Two pathologists, an experienced senior pathologist and a less experienced junior pathologist reevaluated all of the tumor slides stained with hematoxylin and eosin (HE) for the following morphological features and the histological tumor type according to WHO 2003 classification. The morphological features were categorized into 3 groups [14]:

1. Grading factors: Histological grade was assessed using the modified Bloom-Richardson method, in which tubule formation/grade of the tumor, nuclear pleomorphism/atypia (nuclear grade), mitotic count were scored. Mitotic count was performed on Olympus BX51 light microscope, with a graticule at × 40 magnification and in 10 high-power fields (HPFs). Mitotic number was scored as 1 when it was between 0–7, 2 when between 8–14 and 3 when 15 or more.

2. Architectural features of the tumor:

i. Tumor growth pattern was assessed as infiltrative if there was irregular infiltration into the surrounding parenchyma or fat or pushing if the tumor was well circumscribed.

ii. Necrosis with its type was noted as present or absent. Large confluent areas of tumor necrosis with an irregular outline called as geographicnecrosis and the necrosis in the middle of the tumor islands was called as central necrosis.

iii. Stromal lymphocytic response was scored as none, mild (less than 25% of the tumor), moderate (25 to 50% of the tumor) and marked (>50% of the tumor).

iv. Presence or absence of carcinoma in situ was determined.

v. Presence of central scar, defined as the central fibrotic, sclerotic, predomi

3. Cytological features of the tumor cells:

i. Presence of nucleoli were scored as absent or prominent if they were easily visible at low power

ii. Amount of the tumor cell cytoplasm was assessed as scant, moderate or copious according to nuclear-cytoplasm ratio.

iii. Presence of vesicular chromatin pattern was noted.

### Immunocytochemical and immunohistochemical analyses

Immunohistochemical staining was carried out using the DAKO Envision detection kit (Dako, Carpinteria, CA, USA). In brief, paraffin-embedded tissue blocks were sectioned (4 μm-thick), dried, deparaffinized, and rehydrated. Antigen retrieval was performed in a microwave oven for 15 min in 10 mM citrate buffer (pH 6.0). After cells were embedded in 4% neutral formaldehyde for 2 h, PBS with 0.5% Tween-20 was added for 30 min at room temperature. For all samples, endogenous peroxidase activity was blocked with a 3% H_2_O_2_-methanol solution. The slides were blocked with 10% normal goat serum for 10 min and incubated with an appropriately diluted primary antibody overnight at 4°C. The slides were then probed with an HRP-labeled polymer conjugated to an appropriate secondary antibody for 30 min. The antibodies against estrogen receptor (ER), progesterone receptor (PR), HER-2, Ki67, cytokeratin 5/6 (CK5/6) and epidermal growth factor receptor (EGFR) were purchased from Dako (Carpinteria, CA, USA) and the GCS antibody was a gift from Dr. D. Marks (Mayo Clinic Center).

### Interpretation

Staining results were interpreted by a breast pathologist who was blinded to patient outcomes. Tumors with 1% or more positively stained nuclei were considered positive for ER and PR expression [15]. Ki67 staining was determined to be positive when more than 14% of the nuclei were stained. Membranous staining for EGFR and cytoplasmic staining for CK5/6 and HER-2 were scored by counting the number of positively stained cells on the membrane and expressed as a percentage of total tumor cells according to the American Society of Clinical Oncology (ASCO) and the College of American Pathologists (CAP) guidelines using the following categories: 0, no immunostaining; 1+, weak, incomplete membranous staining in any proportion of tumor cells; 2+, complete membranous staining, either non-uniform or weak in at least 10% of tumor cells; and 3+, uniform, intense membranous staining in >30% of tumor cells. HER-2 results were considered positive in cases with 3 + membranous staining of IHC or gene amplification by fluorescence in-situ hybridization (FISH) irrespective of IHC results using the diagnostic criteria described [16].

A dual semi-quantitative scale combining staining intensity and percentage of positive cells was used to evaluate GCS protein staining. The staining intensity was scored as 0 (negative), 1 (weak), 2 (moderate), or 3 (strong). The percentage of positive cells was scored as follows: 0, no staining or staining in <5% of tumor cells; 1, staining in 5% to 25% of cells; 2, staining in 26% to 50% of cells; 3, staining in 51% to 75% of cells; and 4, staining in >75% of cells. For GCS, cytoplasmic staining was considered positive, with an IHC score ≥2 defined as high expression and <2, as low expression [13].

### Fluorescence in-situ hybridization

In cases of HER-2 IHC staining of 2+, fluorescence in-situ hybridization (FISH) analysis was performed manually using the PathVysion HER-2 DNA Probe Kit (Abott, Abott Park, IL, USA) according to the manufacturer’s instructions. In brief, consecutive sections from formalin-fixed, paraffin-embedded tissue blocks were mounted on Probe On Plus microscope slides (Fisher Scientific, Pittsburgh, PA, USA), deparaffinized in xylene, and subsequently rehydrated in ethanol. Sections were then boiled for 10 min in pretreatment solution, incubated with pepsin solution for 10 min, dehydrated in ethanol for 6 min, and finally air-dried. For hybridization, the buffered probe (HER-2/neu and centromere 17) was added to the slide and protected by a coverslip that was sealed with rubber cement. For denaturation, slides were heated to 82°C and incubated overnight at 45°C in a dark, humidified chamber. The rubber cement and coverslip were then removed, and the slides were transferred to stringent wash buffer for 10 min at 65°C. Sections were then dehydrated in ethanol for 6 min and air-dried before being counterstained with 40, 6-diamidino-2- phenylindole (DAPI). Evaluation of signals was carried out using an epifluorescence microscope (Leica, Germany). Counting was carried out according to the manufacturer’s instructions (HER-2/neu gene, orange; centromere 17, green). As recommended by the ASCO/CAP guidelines, an absolute HER-2 gene copy number greater than 6 or HER-2 gene/chromosome 17 copy number ratio higher than 2.2 was considered HER-2 positive. Lymphocytes, fibroblasts, and normal ductal epithelial cells were used as internal controls.

### Definition of breast tumor subtypes

Breast tumor subtypes were defined as follows: luminal A (ER + and/or PR+, HER-2-), luminal B (ER + and/or PR+, HER-2+), HER-2 positive (ER-, PR-, HER-2+), and basal-like (ER-, PR-, HER-2-, Ck5/6 or EGFR+) [14].

### Statistical analysis

Chi-square or Fisher’s exact tests was used to analyze the relationship between the expression of GCS and each histopathological variable. Survival curves were plotted using the Kaplan-Meier method and were compared using the log-rank test. *P-*values less than 0.05 were considered statistically significant. All calculations were performed using the SPSS16.0 for windows statistical software package (SPSS, Chicago, IL, USA).

## Results

### Expression of GCS protein in breast tissue samples

**Figure 1:**
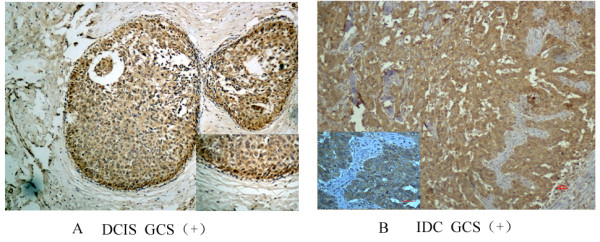
**Immunohistochemical analyses of GCS protein in different sample types.** GCS protein expression detected in all samples by immunohistochemical and cytoplasmic staining was considered positive. Images are representative of two cases that were predominantly positive in DCIS **(A)** or IDC **(B)**, respectively. DCIS-ductal carcinoma in situ; IDC-invasive ductal carcinoma.

The expression of GCS protein was detected in all samples by immunohistochemical staining. Figure 1 show positively and negative staining in invasive ductal breast cancer of breast samples (Figure 1 and Table 3).

**Table 3:** The correlation between GCS and the histopathological variables in 143 cases of invasive breast cancer

		**GCS (+)(104,72.7%)**	**GCS(-) (39, 27.3%)**	***P*-value**
Age	<35	3	5	0.035*
	≥35	101	34	
Tumor stage	T1-2	50	22	0.453
	T3-4	54	17	
Nodal stage	N0	27	15	0.375
	N1-x	23	14	
Histologic grade	Grade I	15	1	0.045
	Grade II-Ш	89	38	
ER	Positive	73	31	0.017
	Negative	19	20	
PR	Positive	69	22	0.271
	Negative	35	17	
HER-2	Positive	22	17	0.007*
	Negative	82	22	
Ki67	<14%	23	7	0.652
	≥14%	81	32	

*P < 0.05.

ER, estrogen receptor; PR, progesterone receptor; HER-2, human epidermal growth factor-2.

### Correlation between GCS expression and clinicopathological parameters

Overall, 72.7% of all the invasive carcinoma samples were positive for GCS (104/143), while only 93.4% of the intraductal proliferative lesions were positive (57/61). The expression level of the GCS protein in the intraductal proliferative lesions was significantly higher than that in the invasive ductal carcinoma (*P* < 0.05).

In the invasive cancers, there was a significant correlation between the GCS upregulation and ER positivity (*P* = 0.017) or HER-2 negativity (*P* = 0.007). We also found that positive rates of GCS expression were higher in grade I (93.75%, 15/16) than those in the grade II–III (70.08%, 89/127) (*P* = 0.045) (Table 3). A higher positive rate of GCS expression was observed in younger patients (aged <35 years) compared with that in older patients (aged ≥35 years).

There was no statistical significance in the relationship between GCS expression and other clinicopathological parameters, including age, tumor size, nodal stage, Ki67 (Table 3).

### Correlation between the GCS expression and the survival

**Figure 2:**
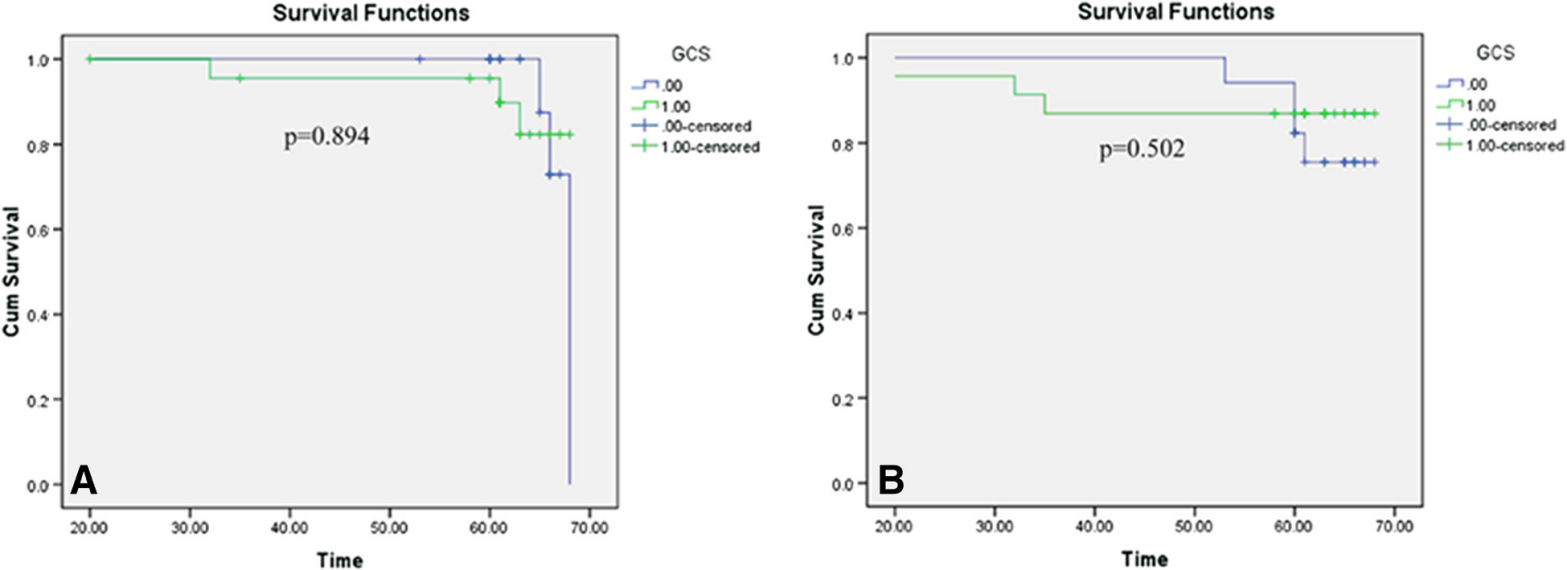
**Patient survival curves.** Kaplan-Meier survival curves for patients with invasive ductal breast carcinoma were calculated according to GCS protein expression status. No correlation was observed between GCS overexpression and **A)** recurrence-free survival or **B)** overall survival.

There was no statistical significance in the relationship between GCS expression and overall survival (Figure 2).

### Correlation between GCS expression and breast tumor molecular subtypes

**Figure 3:**
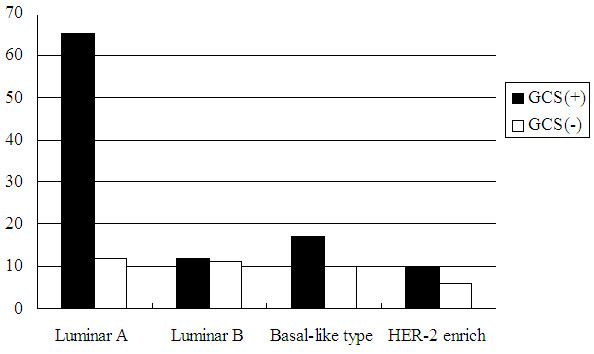
Correlation between GCS expression and breast tumor molecular subtypes.

The positive rate of GCS was highest in luminar A tumors and was lowest in basal-like tumors. However, there was no statistically significant difference in the GCS expression levels between the four breast tumor subtypes (Figure 3).

## Discussion

Breast cancer is one of the most frequent and deadly cancers in women, with an estimated 1,300,000 new cases and 465,000 deaths annually [17]. Multidrug resistance is one of the main impediments to the successful treatment of breast cancer. The mechanisms underlying MDR are complex and overexpression of P-gp is considered to be an important factor.

Recent research has indicated that the expression of P-gp is related to the activity of GCS, an enzyme that glycosylates ceramide and inhibits its proapoptotic activity in cells. Zhang et al. revealed that the expression of the GCS gene in the drug-resistant human breast cancer cell line MCF-7/ADM is higher than that in drug sensitive cells, and that the sensitivity of MCF-7/ADM cells to adriamycin is enhanced by GCS inhibition [18]. Furthermore, GCS expression has been found to confer MDR in many other cancers [19,20]. MDR1 and GCS have been shown to be overexpressed coincidently in several drug-resistant cell lines, a phenomenon that indicates a relationship between these two proteins. In 2010, Liu et al. demonstrated for the first time that GCS upregulates MDR1 expression resulting in the modulation of drug resistance in the ovarian drug-resistant cell line NCI/ADR-RES through the cSrc and beta-catenin signaling pathway [10].

In 2009, microarray analysis of 1,681 breast tumors conducted by Ruckhäberle et al. revealed that GCS mRNA expression was associated with positive ER status, lower histological grading, low Ki67 levels and ErbB2 negativity (*P* < 0.001 for all) [8]. In 2011, Liu et al. detected GCS expression levels in normal tissues and certain cancer tissues. Their results showed that GCS overexpression is highly associated with ER-positive and HER-2-positive breast cancers that have metastasized [12]; however, this was a small study. Our results demonstrated that GCS protein expression was higher in ER-positive samples (*P* < 0.05) (Table 3), which was in accordance with both of these previous studies.

Human epidermal growth factor receptor 2 (HER2) protein, encoded by the oncogene HER2, is amplified in 20–30% of breast cancer cases and is the target of HER2-directed anti-cancer therapies [21]. Our research shows that there was a significant correlation between GCS expression and low HER-2 status in the invasive ductal cancer samples (Table 3), which was in accordance with the study of Ruckhäberle et al., although our observation that GCS protein levels did not correlate with Ki67.

Our study demonstrated a higher positive rate of GCS expression in breast cancer samples from younger patients (aged <35 years) expressed lower levels of GCS protein than older patients (aged ≥35 years) (60% vs. 74.8%, *P* = 0.035). Otherwise, we found that the expression of GCS was higher in the cancer T1-2 than that in the cancer T3-4.

Breast cancer is accounting for 23% (1.38 million) of the total new cancer cases and 14% (458,400) of the total cancer deaths in 2008 worldwide. Metastasis and recurrence severely affect the quality and length of lives of breast cancer patients [22]. Although the study of Liu demonstrated that GCS overexpression is highly associated with ER-positive and HER-2-positive breast cancers that have metastasized [12], our study demonstrated that GCS expression has no correlation with lymph metastasis.

Our data also showed that, in contrast to previous reports, GCS protein expression was much higher in DCIS than that in the invasive ductal cancer.

## Conclusions

In conclusion, our data demonstrate that GCS protein expression is correlated with ER-positive and HER-2 negative breast cancers. Furthermore, intraductal proliferative breast lesions were shown to express significantly higher levels of GCS than that detected in invasive ductal breast cancers.

## Competing interests

All authors declare they have no actual or potential competing financial interest.

## Authors’ contributions

Experiments were conceived and designed by YL and performed by YS, PS and AL. Data were analyzed by DY. The manuscript was written by JL. All authors read and approved the final manuscript.
